# Differential Epigenetic Regulation of Glutamatergic Synapse Pathway in Adults With Prenatal Exposure to Famine

**DOI:** 10.1111/ejn.70195

**Published:** 2025-07-09

**Authors:** Zhen Tan, Weilong Li, Weijing Wang, Dongfeng Zhang, Qihua Tan

**Affiliations:** ^1^ The Second People's Hospital of Lishui Zhejiang China; ^2^ Changchun GeneScience Pharmaceutical Co. Ltd. Changchun China; ^3^ Qingdao University School of Public Health Qingdao China; ^4^ Epidemiology and Biostatistics, Department of Public Health, Faculty of Health Sciences University of Southern Denmark Odense Denmark; ^5^ Unit of Human Genetics, Department of Clinical Research, Faculty of Health Sciences University of Southern Denmark Odense Denmark

**Keywords:** DNA methylation, enrichment analysis, glutamate synapse pathway, prenatal famine, sex‐dependency

## Abstract

It has been hypothesized that poor nutrition during prenatal growth may alter the development of vital organs like the brain, thus “program” predisposition to certain diseases later in life, including mental disorders. Although with support from studies using animal models and epidemiologic observations, the biological aspect of the hypothesis has been rarely studied in humans. Using famine as a natural experiment, we explored the altered DNA methylation patterns in genes of the glutamate synapse pathway in whole blood of adults born during the Chinese famine of 1959–1961. We detected significant patterns of hypomethylation for the whole pathway (*p* = 0.025), for the *GRIA1* gene in the AMPA subunit (*p* = 0.004), for *GRM2* (*p* = 0.023) and *GRM3* (*p* = 0.019) genes in the metabotropic receptor subunit. Our sex‐stratified analysis identified significant enrichment of hypomethylation for the overall pathway (*p* = 0.031), for *GRIA1* genes (*p* = 0.009), *GRIA4* gene (*p* = 0.014), and *GRM3* gene (*p* = 0.031) in females but no significantly enriched pattern in males. Further analysis by location in gene locus found significant enrichment of hypomethylation of the pathway genes in the gene body in sex‐combined (*p* = 0.020) and in female (*p* = 0.026) samples. In conclusion, our epigenetic association analysis found significantly enriched hypomethylation patterns for the glutamate synapse pathway and for genes in subunits of the pathway, which are more pronounced in female than in male samples prenatally exposed to famine.

## Graphical abstract

Using DNA methylation data collected on adults prenatally exposed to the Chinese famine of 1959–1961, this study reports the altered epigenetic regulation in genes of the glutamate synapse pathway in association with early‐life adversity. Our analysis revealed an overall pattern of reduced DNA methylation of the pathway genes that is more pronounced in females than in males. Similar patterns were detected in analyzing genes of specific subunits of the pathway (AMPA and metabotropic receptor subunits). These results are consistent with results from animal studies using brain tissues, suggesting that blood sample‐based epigenetic studies on human natural experiments (e.g., famine) can help to reveal the impact on epigenetic regulation of critical neurological pathways in association with early‐life adversity in humans.

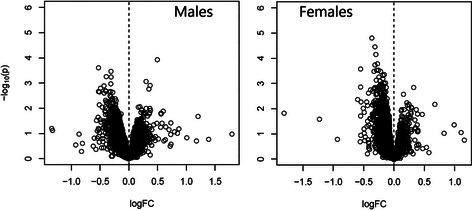

**Abbreviations**
AMPA: α‐amino‐3‐hydroxy‐5‐methyl‐4‐isoxazolepropionic acid
CpG: 5′‐cytosine‐phosphate‐guanine‐3′
EWAS: epigenome‐wide association study
KEGG: Kyoto Encyclopedia of Genes and Genomes
logFC: fold‐change in log scale
NMDA: *N*‐methyl‐d‐aspartate
ROAST: rotation gene set tests
SWAN: subset‐quantile within array normalization
TSS: transcription start sites
UTR: untranslated regions

## 1 Introduction

The fetal origins hypothesis associated with David J. Barker posits that chronic, degenerative conditions of adult health may be triggered by early‐life adverse circumstances, particularly in utero malnutrition (Almond and Currie 2011; Edwards 2017). According to the hypothesis, poor nutrition during prenatal growth may alter the development of vital organs like the brain, thus “program” the predisposition to certain diseases later in life. Due to the strong neuronal proliferation and myelination during neuronal development, the brain is particularly vulnerable to maternal stress. Evidence supporting the hypothesis includes epidemiological studies on the relationship between psychological disorders and exposure to early‐life adversity (Eichenauer and Ehlert 2023; Dana et al. 2019) with data from major famines, for example, the Dutch famine of 1944–1945 (Susser and St Clair 2013) and the Chinese famine of 1959–1961 (Xu et al. 2009; Huang et al. 2013). Among the biological determinants behind the hypothesis, epigenetic mechanisms that act without involving changes in DNA sequences play a central role in the intrauterine modification of gene regulation in response to antenatal maternal adversity (Ryznar et al. 2021).

Glutamate is the major excitatory neurotransmitter at almost all synapses in the central nervous system. Glutamate transmission serves as a crucial target of stress, which introduces changes in glutamate release, transmission, and metabolism to influence cognitive and emotional processing and behavior (Popoli et al. 2011). In an animal model, Brosens et al. (2023) recently reported increased GluA3 content (a subunit encoded by *GRIA3* gene in the family of alpha‐amino‐3‐hydroxy‐5‐methyl‐4‐isoxazole propionate or AMPA receptors) in the hippocampus of early‐life stressed adult male mice at the age of 3 months, suggesting that early‐life adversity affects the development and functioning of hippocampal synapses. Likewise, Al‐Chami et al. (2020) found that early‐life stress prematurely unsilences hippocampal synapses to enhance AMPA receptor functions. Another animal experiment detected increased levels of glutamate *N*‐methyl‐d‐aspartate (NMDA) receptor NR1 (also known as GRIN1) subunit in the prefrontal cortex and the AMPA receptor GluR1 subunit in the hippocampus (Fumagalli et al. 2009), and the metabotropic glutamate (mGlu2/3) receptors in the ventral hippocampus and the prefrontal cortex (Verhaeghe et al. 2022) of adult rats who underwent maternal prenatal stress. Indeed, laboratory studies using the animal model have provided convincing evidence linking early‐life adversity with dysregulation of glutamate transmission. Unfortunately, no study on the topic has been conducted in humans due to ethical reasons.

As an alternative to laboratory studies, famine provides analogous experimental conditions to test the impact of early‐life adversity imposed by maternal famine (malnutrition, stress, etc.) in the offspring. Besides epidemiological investigation, recent epigenome‐wide association studies (EWAS) have identified significant epigenetic changes in adults born during famines. For example, an EWAS on the Dutch famine of 1944–1945 reported altered DNA methylation levels for the *IGF2* gene, a paternally imprinted gene involved in development and growth (Heijmans et al. 2008). Another EWAS on the Chinese Famine of 1959–1961 identified DNA methylation sites involved in neurodevelopment, neuropsychological disorders, and metabolism (Li et al. 2021).

By focusing on the glutamate synapse pathway, this study uses DNA methylation data collected in the EWAS on Chinese famine (Li et al. 2021) and applies statistical testing specifically on the joint differential regulation of the glutamate synapse pathway and related genes. Different from the conventional EWAS that tests each methylation site in the genome while ignoring the functional relatedness of tested sites, our pathway‐based analysis tests the joint methylation patterns of all methylation sites in a pathway presumably with increased statistical power (Wang et al. 2010). Sex‐stratified analysis helps us to detect sex‐dependent patterns of pathway regulation as well as to analyze X‐linked genes in the glutamate synapse pathway, for example, the ionotropic glutamate receptor 3 gene (*GRIA3*). We discuss the impact and limitations of the study and suggest new topics for further research.

## 2 Materials and Methods

### 2.1 The Study Samples

The participants in this study were randomly recruited from a large project on diabetes prevention jointly at Qingdao Center for Disease Control and Prevention (Qingdao CDC) and Qingdao University Medical College, China. The sample includes 79 subjects born during famine (born from Jan. 1, 1959 to Dec. 31, 1961) as the exposed and 105 subjects born after famine (born from Jan. 1, 1963 to Dec. 31, 1964) as the unexposed groups. Participants in the two groups were matched by village, residential, and economic factors. The sampling process excluded subjects with a history of hypertension, diabetes, chronic obstructive pulmonary disease, cancer, stroke, severe mental disorders, tuberculosis, hepatitis, other infectious diseases, and occupational diseases. Whole blood samples were taken from each participant and stored under −80°C for DNA methylation analysis. The study was registered and approved by the Ethics Committee of Qingdao CDC with approval number WDF‐07‐308. Informed consent was obtained from all participants included in the study. The study was conducted under the guidelines set by the Declaration of Helsinki.

### 2.2 DNA Methylation Measurement

DNA methylation levels were measured using the Illumina Infinium MethylationEPIC BeadChip (Illumina) according to standard protocol. A detailed description of the laboratory procedures concerning DNA sample preparation, bisulfite treatment, amplification, hybridization, and so forth can be found elsewhere (Li et al. 2021). For each sample, the array measures DNA methylation levels at 866,091 CpG sites across the genome. The raw DNA methylation data were preprocessed using the R package *minfi* (https://bioconductor.org/packages/release/bioc/html/minfi.html) and underwent a quality control step by probe filtering using the detection *p* value as described in Li et al. (2021) resulting in 863,705 CpGs. The subset‐quantile within array normalization (SWAN) (Maksimovic et al. 2012) implemented in *minfi* was applied for normalizing the array data. After normalization, DNA methylation “beta” value was calculated using Illumina's formula, *β* = *M*/(*M* + *U* + 100), with M and U standing for intensities from the methylated and unmethylated channels at a specific CpG site. Following a data filtering procedure proposed by Wang et al. (2014), we further dropped CpGs with very low methylation variation across samples with standard deviation < 0.02 based on methylation beta values, resulting in 487,229 CpGs for subsequent analysis. The DNA methylation *β* values were finally converted to *M* values by applying the logit transformation as *M* = log_2_(β/(1 − *β*)) to be used for statistical analysis (Du et al. 2010).

### 2.3 Adjusting Blood Cell–Type Compositions

Differences in blood cell composition among the samples could serve as a confounding factor in the statistical analysis of the whole blood‐based DNA methylation data due to cell‐specific methylation patterns. To control for blood cell heterogeneity, we estimated blood cell composition in each individual for six cell types: CD8T, CD4T, natural killer cell, B cell, monocyte, and granulocyte using the method proposed by Houseman et al. (2012) implemented in the R package *minfi*. The estimated proportions of each cell type were included as covariates in statistical testing.

### 2.4 Pathway‐Based Statistical Testing

We first downloaded the annotation of glutamatergic synapse pathway from the KEGG database (pathway ID: hsa04724) at https://www.genome.jp/pathway/hsa04724. The pathway consists of a total of 115 genes, which were mapped to 7347 CpG sites in the processed DNA methylation data, with annotations listed in Table S1. Note that there are 33 CpGs from the *GRIA3* gene located on the X‐chromosome. The rest are from the autosomal chromosomes. Among the 7347 CpGs, 4178 are mapped uniquely to the gene body, 1747 uniquely to promoters (including specific mapping of 598 to TSS1500, 333 to TSS200 and 672 to 5′UTR), 73 uniquely to 1stExon, and 197 uniquely to 3′UTR.

With the mapped pathway CpGs, we first performed an association analysis of each CpG site with prenatal famine exposure adjusting for age, sex, and blood cell composition using the *lmFit()* function for linear regression with the *M* value and the *eBayes()* function for empirical Bayes moderation of the test statistics implemented in the *limma* package of Bioconductor version 3.9. The statistical model included age for adjustment because the exposure group is on average 4 years older than the unexposed group. Next, we moved on with jointly testing the differential methylation pattern of the whole pathway by pathway enrichment analysis using the Rotation Gene Set Tests (ROAST) (Wu et al. 2010) implemented in the *limma* package. ROAST provides a self‐contained and statistically rigorous gene set test that introduces residual space rotation for multivariate regression instead of permutation while allowing for gene‐wise correlation. The method applies to complex experimental designs and to studies with small numbers of replicates. A similar regression model as for the association testing on single CpGs is introduced in the design matrix which regresses DNA methylation M value on prenatal exposure to famine (yes = 1, no = 0) adjusting for other covariates including age, sex, and blood cell composition estimates. The method is the only pathway‐based test that incorporates both up and down regulated patterns simultaneously (Giner and Smyth 2016). For each regulation pattern (e.g., hyper or hypomethylation), the method estimates an active proportion as the percentage of CpGs with a log scaled fold change (logFC) of DNAm between the exposed and the unexposed more than one standard error above zero.

## 3 Results

### 3.1 Single‐Site Testing on Differential Methylation in the Glutamatergic Synapse Pathway

From single‐site association testing, we found 644 CpGs with *p* < 0.05 (8.8%), 165 CpGs with *p* < 0.01 (2.26%) and 21 CpGs with *p* < 0.001 (0.29%), which are all higher than the randomly expected proportions (Table S2). One CpG site cg11758280 on chromosome 20 at 9153867 bp is hypermethylated with the highest significance of *p* = 1.36e−6. Another site cg20083839 on chromosome 20 at 57485940 bp is hypomethylated with *p* = 8.44e−6. These observations could indicate a broad involvement of differential DNAm in the pathway genes.

### 3.2 Enrichment Testing on the Glutamatergic Synapse Pathway

**FIGURE 1:**
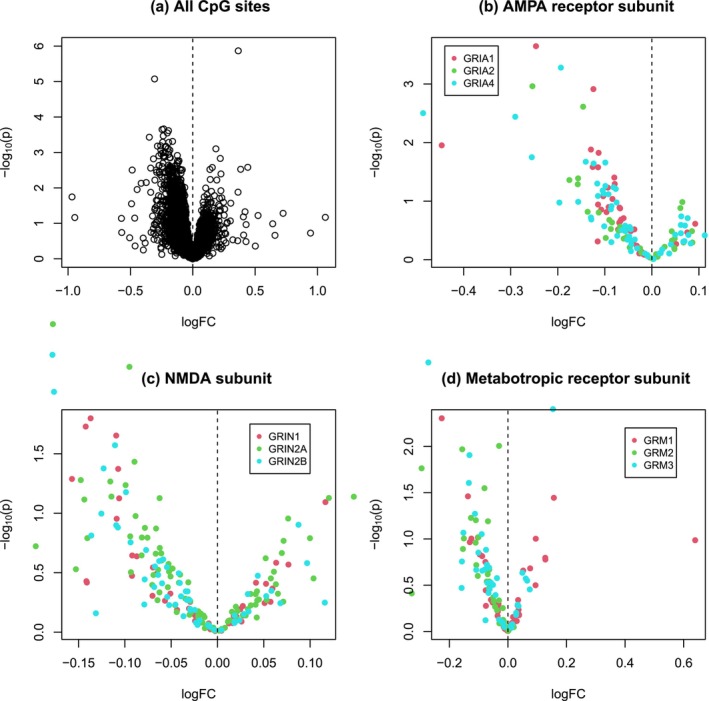
Volcano plots displaying the differential DNA methylation patterns of CpG sites of the glutamate synapse pathway in all genes of the pathway (a), genes of the AMPA receptor subunit (b), genes of the NMDA subunit (c), and genes of the metabotropic receptor subunit (d), with *Y*‐axis for the minus log‐scaled (based 10) *p* value of each CpG and the *X*‐axis for the corresponding fold change (FC) of *M* value in log scale indicating increased (logFC > 0) or decreased (logFC < 0) methylation in the exposure group.

**FIGURE 2:**
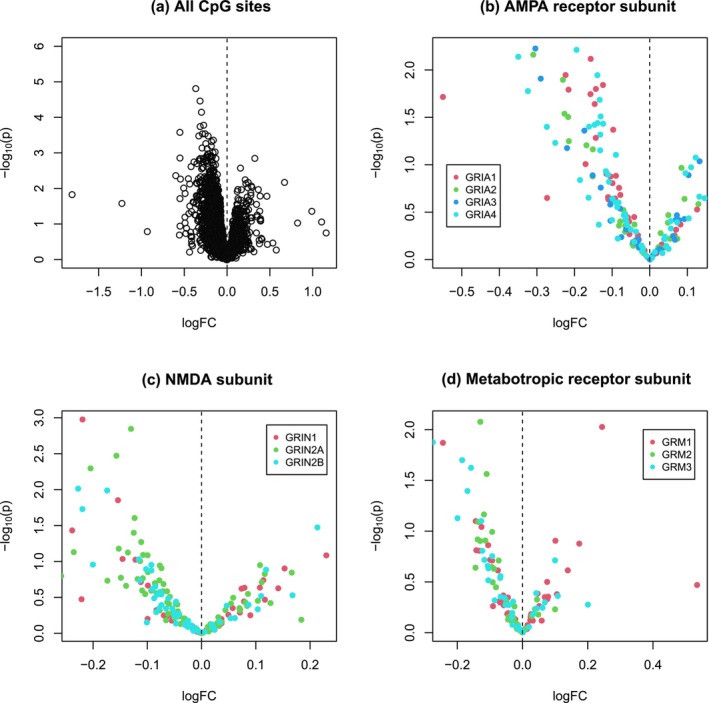
Volcano plots showing the female sample differential DNA methylation patterns of CpG sites of the glutamate synapse pathway in all genes of the pathway (a), genes of the AMPA receptor subunit (b), genes of the NMDA subunit (c), and genes of the metabotropic receptor subunit (d), with *Y*‐axis for the minus log‐scaled (based 10) *p* value of each CpG and the *X*‐axis for the corresponding fold change (FC) of *M* value in log scale indicatng increased (logFC > 0) or decreased (logFC < 0) methylation in the exposure group.

**FIGURE 3:**
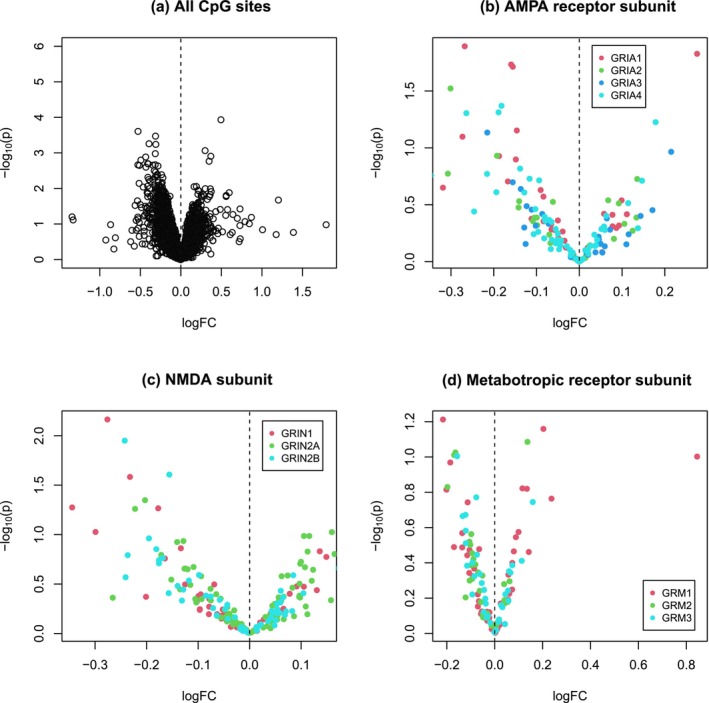
Volcano plots showing the male sample differential DNA methylation patterns of CpG sites of the glutamate synapse pathway in all genes of the pathway (a), genes of the AMPA receptor subunit (b), genes of the NMDA subunit (c), and genes of the metabotropic receptor subunit (d), with *Y*‐axis for the minus log‐scaled (based 10) *p* value of each CpG and the *X*‐axis for the corresponding fold change (FC) of *M* value in log scale indicating increased (logFC > 0) or decreased (logFC < 0) methylation in the exposure group.

By applying ROAST for gene‐set enrichment analysis, we found that the pathway is in general significantly down or hypomethylated (*p* = 0.025, active proportion 45%) (Table 1). The pattern of hypomethylation is illustrated in the scatter plot of Figure 1a, where there are more hypomethylated than hypermethylated CpGs among the top significant sites. We further performed sex‐stratified pathway analysis in male and female samples separately. As shown in Table 1, we only detected a significant hypomethylation pattern of the pathway in females (*p* = 0.031, active proportion 38%) but not in males, suggesting sex‐dependent epigenetic regulation of the pathway by maternal famine. The differential hypomethylation pattern is supported by the single‐site based association testing for females (Figure 2a, Table S3). Although it seems there are slightly more hypomethylated than hypermethylated sites in the pathway (Figure 3a, Table S4), no statistically significant result is obtained for male samples (Table 1).

**TABLE 1:** Results of pathway enrichment tests.

Enrichment pattern	Active proportion	*p* value
All samples		
Down	0.199	**0.025**
Up	0.028	0.975
Up or down	0.199	0.050
Mixed	0.227	0.100
Females		
Down	0.199	**0.031**
Up	0.027	0.969
Up or down	0.199	0.063
Mixed	0.226	0.119
Males		
Down	0.086	0.310
Up	0.044	0.691
Up or down	0.086	0.619
Mixed	0.130	0.619

### 3.3 Enrichment Testing on AMPA, NMDA, and Metabotropic Receptor Subunits

We moved on with enrichment testing of differential methylation patterns in genes belonging to specific subunits of the pathway focusing on AMPA, NMDA, and metabotropic receptor subunits. The AMPA subunit includes 4 genes, *GRIA1*, *GRIA2*, *GRIA3*, and *GRIA4*. Table 2 presents the enrichment results for the four genes, with only *GRIA1* significantly enriched by a pattern of hypomethylation in the sex‐combined analysis (*p* = 0.004, active proportion 45%). No significant result was found for the other genes, although *GRIA4* showed an enrichment pattern of hypomethylation with *p* = 0.069. Note the sex‐combined analysis was not able to include *GRIA3* on the X‐chromosome because of X‐chromosome inactivation in females. In the sex‐stratified analysis, both *GRIA1* and *GRIA4* were significantly enriched for hypomethylation in female samples (*p* = 0.009 for *GRIA1*, *p* = 0.014 for *GRIA4*) with active proportions over 34%. No significant enrichment was found in male samples.

**TABLE 2:** Enrichment of genes in AMPA receptor subunit.

Enrichment pattern	All samples		Female		Male	
	Active proportion	*p* value	Active proportion	*p*	Active proportion	*p* value
GRIA1						
Down	0.450	**0.004**	0.375	**0.009**	0.175	0.139
Up	0.000	0.996	0.000	0.991	0.025	0.862
Up or down	0.450	0.008	0.375	0.019	0.175	0.277
Mixed	0.450	0.008	0.375	0.015	0.200	0.261
GRIA2						
Down	0.212	0.069	0.273	0.056	0.061	0.242
Up	0.061	0.931	0.030	0.944	0.000	0.758
Up or down	0.212	0.139	0.273	0.113	0.061	0.485
Mixed	0.273	0.060	0.303	0.034	0.061	0.872
GRIA3						
Down			0.152	0.175	0.030	0.527
Up			0.061	0.825	0.061	0.473
Up or down			0.152	0.350	0.061	0.947
Mixed			0.212	0.268	0.091	0.945
GRIA4						
Down	0.212	0.069	0.345	**0.014**	0.086	0.163
Up	0.061	0.931	0.052	0.986	0.034	0.837
Up or down	0.212	0.139	0.345	0.028	0.086	0.327
Mixed	0.273	0.060	0.397	0.007	0.121	0.614

The NMDA subunit consists of *GRIN1*, *GRIN2A*, and *GRIN2B*. No significant enrichment was found in either sex‐combined or sex‐stratified analysis (Table S5). Of the three genes in the metabotropic receptor subunit, *GRM1*, *GRM2*, and *GRM3*, significant enrichments of hypomethylation were detected for *GRM2* in sex‐combined analysis (*p* = 0.023, active proportion 26%), and for *GRM3* in sex‐combined (*p* = 0.019, active proportion 21%) and in female samples (*p* = 0.031, active proportion 21%) (Table 3). Again, no significant enrichment was detected in male samples.

**TABLE 3:** Enrichment of genes in metabotropic receptor subunit.

Enrichment pattern	All samples		Female		Male	
	Active proportion	*p* value	Active proportion	*p* value	Active proportion	*p* value
GRM1						
Down	0.137	0.376	0.118	0.349	0.059	0.442
Up	0.059	0.624	0.059	0.651	0.078	0.558
Up or down	0.137	0.753	0.118	0.698	0.078	0.884
Mixed	0.196	0.657	0.176	0.625	0.137	0.865
GRM2						
Down	0.257	**0.023**	0.229	0.079	0.143	0.132
Up	0.000	0.978	0.000	0.921	0.029	0.868
Up or down	0.257	0.045	0.229	0.158	0.143	0.264
Mixed	0.257	0.253	0.229	0.409	0.171	0.505
GRM3						
Down	0.205	**0.019**	0.205	**0.031**	0.077	0.205
Up	0.000	0.982	0.000	0.969	0.026	0.795
Up or down	0.205	0.037	0.205	0.062	0.077	0.410
Mixed	0.205	0.077	0.205	0.188	0.103	0.520

### 3.4 Enrichment Testing on the Glutamatergic Synapse Pathway by Location of Methylation Sites

Considering the fact that DNA methylation can either repress or activate gene expression, depending on its precise location within the genetic locus, we further performed enrichment analysis on methylation patterns of the pathway by location of the CpG sites divided by CpGs mapped uniquely to gene body, to promoters, and CpGs mapped to a mixture of different locations of different genes (heterogeneous). We found significant enrichment of a hypomethylation pattern in the gene body in sex‐combined samples (*p* = 0.020, active proportion 23%) and in female samples (*p* = 0.026, active proportion 23%) (Table 4). CpGs mapped to heterogeneous locus locations also display an enriched pattern of hypomethylation but only with a borderline significance (*p* = 0.047, ative proportion 17%). Surprisingly, we failed to detect any significantly enriched DNA methylation pattern for CpGs solely mapped to the promoter region.

**TABLE 4:** Enrichment patterns by location of CpGs.

Enrichment pattern	All samples		Female		Male	
	Active proportion	*p* value	Active proportion	*p* value	Active proportion	*p* value
Gene body						
Down	0.231	**0.020**	0.226	**0.026**	0.092	0.275
Up	0.019	0.980	0.021	0.975	0.038	0.726
Up or down	0.231	0.040	0.226	0.051	0.092	0.549
Mixed	0.250	0.068	0.247	0.052	0.129	0.661
Promoter						
Down	0.147	0.166	0.161	0.095	0.067	0.539
Up	0.049	0.834	0.040	0.905	0.061	0.461
Up or down	0.147	0.333	0.161	0.191	0.067	0.922
Mixed	0.195	0.200	0.202	0.253	0.128	0.570
Mixture						
Down	0.168	**0.047**	0.162	0.069	0.092	0.244
Up	0.032	0.954	0.032	0.932	0.045	0.756
Up or down	0.168	0.093	0.162	0.137	0.092	0.488
Mixed	0.200	0.231	0.194	0.275	0.137	0.473

## 4 Discussion

The association of early‐life adversity with adult psychiatric disorders has been revealed by both epidemiological studies on humans (Dana et al. 2019) and studies using animal models (DeCapo et al. 2019). It is critical to study the biological mechanisms behind the observed association both for exploring disease aetiology and for prevention and treatment of related disorders. Current studies on the regulation of glutamatergic synapse pathway genes by early‐life adversity have been, up to date, limited to the animal model. This study, for the first time, extends the analysis to human samples by (1) using famine as a natural experiment and (2) applying bioinformatics tools for analyzing the overall pathway as well as specific genes of the pathway. Significantly enriched hypomethylation patterns have been detected for the overall pathway and for specific genes consisting of subunits of the pathway, with a sex‐dependent effect of maternal famine that specifically affects female offspring. The sex‐dependent results seem to be in agreement with epidemiological observations that reported a higher risk of schizophrenia in females than in males born in mid‐adulthood during the Chinese Famine (Wang and Zhang 2017). Likewise, Zhou et al. (2023) also reported a significant correlation of birth seasonality with adulthood depressive symptoms, which affect females more than males. By focusing on epigenetic regulation patterns of genes in the glutamatergic synapse pathway, this study explores the biological basis of the epidemiological findings in humans.

Prenatal neuroplasticity allows neurons to regenerate anatomically and functionally for reprogramming brain development in accordance with environmental stimuli such as maternal stress. Results from the current study on adults exposed to prenatal famine suggest that early‐life adversity could exert significant long‐lasting epigenetic modification of important neurotransmission pathways. Indeed, the samples included in this study are currently free from diagnosed neuropsychiatric disorders. However, the detected significant epigenetic alteration in the important neurotransmission pathways could impact their susceptibility to mental diseases. The observation calls for supportive and intervention strategies to promote the mental health of the exposed population.

In a recent study, Zeng et al. (2023) reported the long‐term effect of prenatal famine on DNA methylation of genes in the serotonin receptor signalling pathway. Their analysis identified a significant pattern of reduced DNA methylation in sex combined samples but which is only significant in females but not in males. Similar pattern of hypomethylation was also observed for specific genes of the serotonin receptor signalling pathway in *HTR2A* and *HTR2C*, again in females but not in males. Interestingly, the enriched hypomethylation was observed mainly in the promoter regions of the genes. In the current study, however, enrichment of hypomethylation was mainly detected in gene body. It is well known that DNA methylation in promoters surpress gene expression while methylation in gene body is positively correlated with expression (Yang et al. 2014). The neurotransmitter serotonin can colocalize in the same nerve terminal with other neurotransmitters and reciprocal interactions take place to mediate respectively excitatory and inhibitory signals in the central nerves system (Ciranna 2006). The different locations of the detected enrichment of hypomethylation patterns in serontonin and glutamate neurotransmitter genes in association with prenatal famine may indicate the reciprocal interaction between serotonin and glutamate involved in psychosis pathophysiology (de Bartolomeis et al. 2013).

The Illumina EPIC probes are distributed at promoters (54%), followed by gene bodies (30%) and other regions (16%) (Pidsley et al. 2016). Although the array used in this study is dominated by probes located at promoters, the CpGs matched to the glutamatergic synapse pathway genes are mainly uniquely annotated to gene body regions, followed by promoters. This difference in CpG distribution could have contributed to the significant detection of enrichment patterns in the gene body. It would be interesting to apply a similar analysis to whole‐genome DNA methylation sequencing data to avoid bias in probe distribution by array techniques.

It is necessary to point out that current analysis on glutamatergic synapse pathway genes was conducted on DNA methylation data collected from the whole blood, which is different from published animal studies that looked at brain tissues. The difficulty of obtaining brain tissue from human samples has stumped progress towards understanding the molecular mechanisms of complex psychiatric disorders. By comparing cross‐tissue DNA methylation patterns, Mendonça et al. (2024) reported recently that blood DNA can be used as a surrogate of brain tissue to analyze changes in CpG methylation level in neurodegenerative diseases. The consistent findings from our human study using whole blood with animal studies using brain tissues support their observation. Even though we need to emphasize that our conclusions are simply based on statistical correlations, which may not be biologically causal. Nevertheless, our results show that measuring the DNA methylation patterns in blood samples from adults exposed to early‐life adversities (e.g., famine) opens an opportunity for studying the associated epigenetic regulation patterns characterizing analogous molecular mechanisms of the brain tissues in human samples.

## Author Contributions

Q.T. and Z.D. designed the study. Z.D. and W.W. collected data. W.L., Q.T., and Z.T. performed analysis. Q.T., Z.D., W.L., and Z.T. contributed to data interpretations. Z.T. and Q.T. drafted and revised the manuscript.

## Conflicts of Interest

The authors declare no conflicts of interest.

## Peer Review

The peer review history for this article is available at https://www.webofscience.com/api/gateway/wos/peer‐review/10.1111/ejn.70195.

## Supporting information


**Table S1.** Supporting Information

**Table S2.** Supporting Information

**Table S3.** Supporting Information

**Table S4.** Supporting Information

**Table S5.** Supporting Information

## Data Availability

According to current Danish and EU legislations, transfer and sharing of individual‐level data require prior approval from the Danish Data Protection Agency. Our present local data protection rules do not allow individual‐level data to be shared in public databases. For these reasons, the raw data cannot be deposited in a public database. However, we welcome any enquiries regarding collaboration and individual requests for data sharing. Requests can be directed to Qihua Tan at qtan@health.sdu.dk.
